# Clinical decision-making for uveal melanoma radiotherapy: comparative performance of experienced radiation oncologists and leading generative AI models

**DOI:** 10.3389/fonc.2025.1605916

**Published:** 2025-08-14

**Authors:** Xing Wang, Peng Wang

**Affiliations:** Department of Ophthalmology, The First Affiliated Hospital of Chongqing Medical University, Chongqing Key Laboratory for the Prevention and Treatment of Major Blinding Eye Diseases, Chongqing, China

**Keywords:** uveal melanoma, radiotherapy, clinical decision-making, generative AI, oncology

## Abstract

**Background:**

Uveal melanoma is the most common primary intraocular malignancy in adults, yet radiotherapy decision-making for this disease often remains complex and variable. Although emerging generative AI models have shown promise in synthesizing vast clinical information, few studies have systematically compared their performance against experienced radiation oncologists in this specialized domain. This study examined the comparative accuracy of three leading generative AI models and experienced radiation oncologists in guideline-based clinical decision-making for uveal melanoma.

**Methods:**

A structured, 20-question examination reflecting standard radiotherapy guidelines was developed. Fifty radiation oncologists, each with 10–15 years of experience, completed an open-book exam following a 15-day standardized review. Meanwhile, Grok 3 (Think), Gemini 2.0 Flash Thinking, and Open ai o1 pro were each tested through 10 independent chat sessions. Twelve recognized experts in uveal melanoma, blinded to the source of each submission, scored all answer sets. Kruskal–Wallis tests with *post hoc* comparisons were conducted to evaluate group-level differences in total and domain-specific performance.

**Results:**

Of the 80 total sets (50 from oncologists, 30 from AI), Open ai o1 pro achieved the highest mean total score (98.0 ± 1.9), followed by oncologists (91.5 ± 3.2), Grok 3 (82.3 ± 2.1), and Gemini 2.0 (74.2 ± 3.4). Statistically significant differences emerged across all domains, with human experts particularly excelling in treatment selection but still trailing Open ai o1 pro overall. Completion time was significantly shorter for the AI models compared with oncologists.

**Conclusion:**

These findings suggest that advanced generative AI can exceed expert-level performance in certain aspects of radiotherapy decision-making for uveal melanoma. Although AI may expedite clinical workflows and offer highly accurate guidance, human judgment remains indispensable for nuanced patient care.

## 1 Introduction

Uveal melanoma represents the most common primary intraocular malignancy in adults, characterized by significant heterogeneity in clinical presentation, prognosis, and treatment responses (1–4). Radiotherapy, encompassing modalities such as plaque brachytherapy and proton beam therapy, remains a central component of definitive management, aimed at achieving effective tumor control while maximizing preservation of visual function and quality of life (5–10). Optimal clinical decision-making in radiotherapy for uveal melanoma requires integration of complex patient- and tumor-specific variables, meticulous staging assessments, precise radiation planning, and careful evaluation of potential treatment-related morbidity (11–15). Despite established guidelines and consensus recommendations, variability persists in therapeutic approaches among clinicians, reflecting the inherently nuanced nature of individualized patient care.

With recent advances in computational capabilities and artificial intelligence, generative AI models, particularly large language models, have increasingly been explored as potential tools to assist clinical decision-making across various medical disciplines (16–21). Such models, trained on vast corpora of medical literature and clinical guidelines, hold promise for synthesizing information rapidly and consistently, potentially supporting clinicians in complex diagnostic and treatment selection processes (22–25). Yet, the effectiveness and accuracy of generative AI in clinical decision-making remain subjects of ongoing research and debate, especially in highly specialized fields such as radiation oncology, where nuanced judgment, detailed anatomical considerations, and individualized patient assessments are paramount. While previous studies have compared AI-assisted decision-making with human experts across diverse clinical contexts, few rigorous investigations have systematically examined the comparative performance of leading generative AI models against experienced radiation oncologists specifically within the domain of uveal melanoma radiotherapy (26–29). Understanding how state-of-the-art generative AI systems perform relative to seasoned specialists may offer valuable insights into their potential clinical utility, help delineate areas where AI may complement or enhance human expertise, and identify domains where clinical judgment remains indispensable. Retinoblastoma, while also governed by detailed protocols, was not selected because its modern management pivotally integrates systemic chemotherapy, intra−arterial infusions, and intravitreal agents—modalities that lie largely outside a radiotherapy decision matrix. Uveal melanoma, by contrast, remains radiotherapy−centric and is therefore a purer proving ground for assessing how language models handle guideline−dense radiation questions without confounding systemic−therapy variables.

Therefore, this study was designed to comparatively evaluate the decision-making performance of highly experienced radiation oncologists and prominent generative AI models in guideline-based clinical assessments for uveal melanoma radiotherapy. By meticulously analyzing their responses to a structured examination encompassing critical domains of diagnosis, staging, treatment selection, and radiation planning, we aimed to elucidate the capabilities, strengths, and limitations inherent to human expertise and generative AI. Through this comparative analysis, our objective was to inform clinical practices, foster deeper collaboration between clinicians and emerging AI tools, and ultimately contribute to optimizing therapeutic decision-making processes and outcomes for patients with uveal melanoma. The three generative AI models were purposefully chosen because each embodies a different large-language-model training philosophy. Grok 3 (Think) is fine-tuned chiefly on biomedical literature, Gemini 2.0 Flash Thinking blends instruction-following reinforcement with real-time web snapshots, and Open ai o1 pro employs a reinforced-self-training loop on a broad general-domain corpus with subsequent medical-domain alignment. Comparing these architectures—each previously reported to perform in the upper quartile of benchmark medical-QA leaderboards—allowed us to test whether radiotherapy accuracy is driven primarily by model scale, biomedical fine-tuning, or real-time web augmentation.

## 2 Methods

### 2.1 Study design

A guideline-based clinical examination, focusing on radiotherapy decision-making for patients with uveal melanoma, was developed to evaluate and compare performance between experienced radiation oncologists and leading generative AI models. The exam comprised 20 case−based questions that probed radiotherapy−specific decision−making; scenarios involving adjuvant systemic therapy were deliberately excluded because current agents (e.g., immune checkpoint inhibitors, tebentafusp) are governed by distinct medical−oncology guidelines and do not alter primary radiation−planning parameters under study. The examination papers are shown in **Appendix Table 1** (30–40). Each question was awarded 5 points, yielding a total possible score of 100. Because the participating large language models primarily operate in English, the exam paper was prepared in English. Although the participating radiation oncologists were native Chinese speakers, their documented proficiency in English (IELTS ≥7 or TOEFL ≥110) assured the ability to complete the exam in this language. They were permitted to use any translation tools or reference materials other than large language models; nevertheless, we acknowledge that writing in a second language may have modestly prolonged their response time.

### 2.2 Exam development

The exam content was created in strict adherence to established guidelines and expert consensus regarding uveal melanoma radiotherapy. A thorough literature review of relevant guidelines was conducted, and essential topics——spanning diagnosis, tumor staging, treatment selection, radiation planning were incorporated into the final 20 case-based questions. Each question was allocated a maximum of 5 points—with diagnosis, staging, treatment selection, and radiation planning each contributing an equal 25−point quadrant—because leading consensus documents (ABS, ESTRO−ASTRO, NCCN) devote comparable emphasis to these four pillars, and pilot testing showed that unequal weights inflated variance without improving construct validity; partial credit was still awarded for nuanced or partially correct answers. A brief justification for equal weighting was provided to participants in advance to ensure transparency and discourage domain−selective study strategies.

### 2.3 Training materials and participant recruitment

A dedicated set of review materials was compiled, delivered as: 08:00-09:30 didactic lectures on guideline updates; 10:00-12:00 software-assisted contouring labs; 14:00-16:30 small-group case simulations; and 19:00-20:30 moderated journal-club discussions, each recorded and accompanied by formative quizzes. Learning objectives and reading list. The 15-day course targeted six objectives: (i) apply 2024−update AJCC staging, (ii) select modality-specific dose prescriptions, (iii) contour ocular critical structures reproducibly, (iv) recognize imaging red−flags denoting high-risk nevi, (v) counsel on radiation−induced toxicities, and (vi) integrate systemic-therapy indications. Required readings comprised the 2024 ABS brachytherapy guideline, ESTRO proton−beam consensus, the NCCN uveal-melanoma pathway, and three sentinel meta−analyses. Daily formative quizzes (20 multiple-choice items and one short−answer vignette) were marked automatically; ≥80 % was required to advance, ensuring baseline homogenization. Fifty radiation oncologists, all holding a doctoral degree and possessing 10–15 years of professional experience, were recruited. This number was selected *a priori* because a power analysis (α = 0.05, two-tailed; effect size d = 0.65 derived from our pilot study) indicated that ≥48 human participants would yield 90% power to detect a 5-point difference in total score; two additional clinicians were enrolled to compensate for potential attrition. Each had published at least three first-author or corresponding-author articles in SCI-indexed journals with an impact factor >5 within the last five years and had reached a total citation count exceeding 200 with an h-index above 10 by March 1, 2025. These participants completed the focused 15-day review course before taking the exam. To promote uniform baseline knowledge, the 15-day course combined didactic morning lectures delivered by senior ocular oncologists, small-group afternoon workshops featuring live treatment-planning software demonstrations, and nightly guided journal-club discussions. Attendance was mandatory and logged; daily formative quizzes gauged mastery of the learning objectives and fed back personalized study tips. All 50 oncologists practice in tertiary−care academic hospitals that adhere to national radiotherapy guidelines; 38 (76 %) routinely manage ocular tumors—including uveal melanoma—at a median volume of 18 cases per year, whereas the remaining 12 focus primarily on broader head−and−neck disease but attend ocular−oncology multidisciplinary rounds at least quarterly; 46 (92 %) also reported only cursory hands−on exposure (<5 h) to large language−model chatbots prior to enrolment, minimizing confounding by pre−existing AI familiarity. The Grok 3 and Gemini 2.0 sandboxes were forced into “no−network” offline snapshots behind an institutional firewall, and Open ai o1 pro was queried in a research tenant with external API calls disabled; thus none of the models could initiate real−time web searches during inference. Completion−time correlation. Pearson testing showed no significant correlation between individual oncologists’ completion time and total score (r = -0.07, p = 0.59), suggesting that slower pacing did not confer an accuracy advantage but may reflect higher cognitive-load or translation overhead. Safeguards against hallucination or unsafe advice remain pivotal before embedding generative models into clinical workflows. We advocate model−version locking, provenance logging, dose−constraint hard−checks, and a “human−in−the−loop veto” that blocks any recommendation triggering preset safety keywords (e.g., doses exceeding optic−nerve tolerance). Prospective trials should report hallucination rates per treatment−plan component and monitor near−misses to strengthen governance. Bilingual workflow assurance. All English prompts were back−translated into Mandarin by two independent medical translators; discrepancies were adjudicated in consensus meetings until semantic equivalence was confirmed. During the examination, clinicians could cross−check any phrase with browser−based translation, and a post−hoc audit of 120 random responses found no wording ambiguities that altered grading.

### 2.4 Testing procedure

Following the training period, the 50 radiation oncologists were invited to participate in an open−book examination — a deliberate choice because real−world radiotherapy decisions are almost never made from unaided memory but rather alongside guidelines, dose−constraint tables, and multidisciplinary notes. Each individual was allowed up to 5 hours to answer all 20 questions (total 100 points), a window chosen to approximate the reflection time and guideline checks that typically occur during multidisciplinary chart reviews. All reference materials (printed or electronic), plus browser−based translation tools, were permitted, whereas large−language−model assistance and live web queries were explicitly disabled to isolate human performance; this design mirrors routine tumor−board discussions yet intentionally devotes less weight to the acute time−pressure that closed−book or oral boards impose. In parallel, three generative AI models—Grok 3 (Think, build v3.1-2025-02-08), Gemini 2.0 Flash Thinking (release 2025-03-12), and Open ai o1 pro (model-snapshot 2025-02-28)—were each prompted; the same frozen version was used for all ten sessions per model to ensure reproducibility.—were each prompted with the same set of 20 questions on 10 independent chat dialogs, a number chosen for feasibility and because simulation–based power calculations showed that ten replicates per model stabilized the mean score within ±1.5 points, leading to a total of 30 AI-generated answer sets. To ensure anonymity and objectivity, 30 doctors (who did not participate in the exam) meticulously transcribed the AI−generated answers by hand, thereby stripping machine−generated typography, lexical idiosyncrasies, or systematic punctuation patterns that can betray an AI origin; every sheet was re−copied and double-checked, and the twelve examiners received no training in forensic linguistics so as to avoid cue-seeking bias. Patient−identifier handling. All clinical vignettes were synthetically constructed or fully de−identified under GDPR Article 26 and the 2024 PRC Personal Information Protection Law; no direct or indirect identifiers were stored in prompt logs or examiner worksheets.

### 2.5 Accuracy assessment

Twelve recognized experts in uveal melanoma were invited to score the completed exams. Six of these experts were senior chief physicians with more than 20 years of clinical experience, stationed at top-tier tertiary hospitals, each having completed over 300 related surgeries. The other six were tenured professors with over 20 years of teaching experience in the uveal melanoma domain, employed at West China Hospital of Sichuan University with h-indices above 30. Every examiner graded each of the 80 anonymized answer sets (50 from radiation oncologists, 30 from the AI models). For each of the 20 questions, the highest and lowest marks were discarded, and the remaining scores were averaged to obtain the final score. This method minimized the influence of extreme ratings and allowed for a fairer representation of the true performance.

### 2.6 Statistical analysis

All statistical analyses were performed using SPSS version 26. Data for the total exam scores were typically presented as means ± standard deviations if normally distributed or as medians (interquartile ranges) if the distribution was skewed. The Kruskal–Wallis H test was used to compare median scores across the different AI models and the human participants. *Post hoc* pairwise tests were conducted if a significant difference was observed. A p-value below 0.05 was regarded as indicating statistical significance. Fleiss’ Kappa was calculated to assess the consistency among the 12 experts’ ratings, with values ranging from 0 to 1.0, where 0 indicates no agreement and 1.0 represents perfect agreement.

## 3 Results

### 3.1 Participant characteristics of radiation oncologists

A total of 50 experienced radiation oncologists participated in this study, all of whom met the eligibility criteria detailed in the Methods. The mean age was 48.6 years (SD = 7.4), and 58% were male. They had an average of 15.8 years (SD = 5.2) of professional experience in radiation oncology. All possessed doctoral degrees, had published at least three first- or corresponding-author SCI-indexed articles (impact factor >5) in the past five years, and demonstrated a mean h-index of 16.5 (SD = 4.5). **Table 1** presents demographic and professional characteristics of these oncologists.

**Table 1:** Baseline characteristics of the 50 radiation oncologists.

Variable	Value
Age, years (mean ± SD)	48.6 ± 7.4
Sex, n (%)	Male: 29 (58%); Female: 21 (42%)
Professional experience, years (mean ± SD)	15.8 ± 5.2
h-index (mean ± SD)	16.5 ± 4.5
Number of SCI publications (median, IQR)	10 (5–28)
Training period completed (yes/no)	Yes: 50 (100%); No: 0 (0%)
Familiarity with English exam paper	IELTS ≥7 or TOEFL ≥110, all confirmed

IELTS, International English Language Testing System; TOEFL, Test of English as a Foreign Language.

### 3.2 Overall exam scores for radiation oncologists and AI models

**Figure 1:**
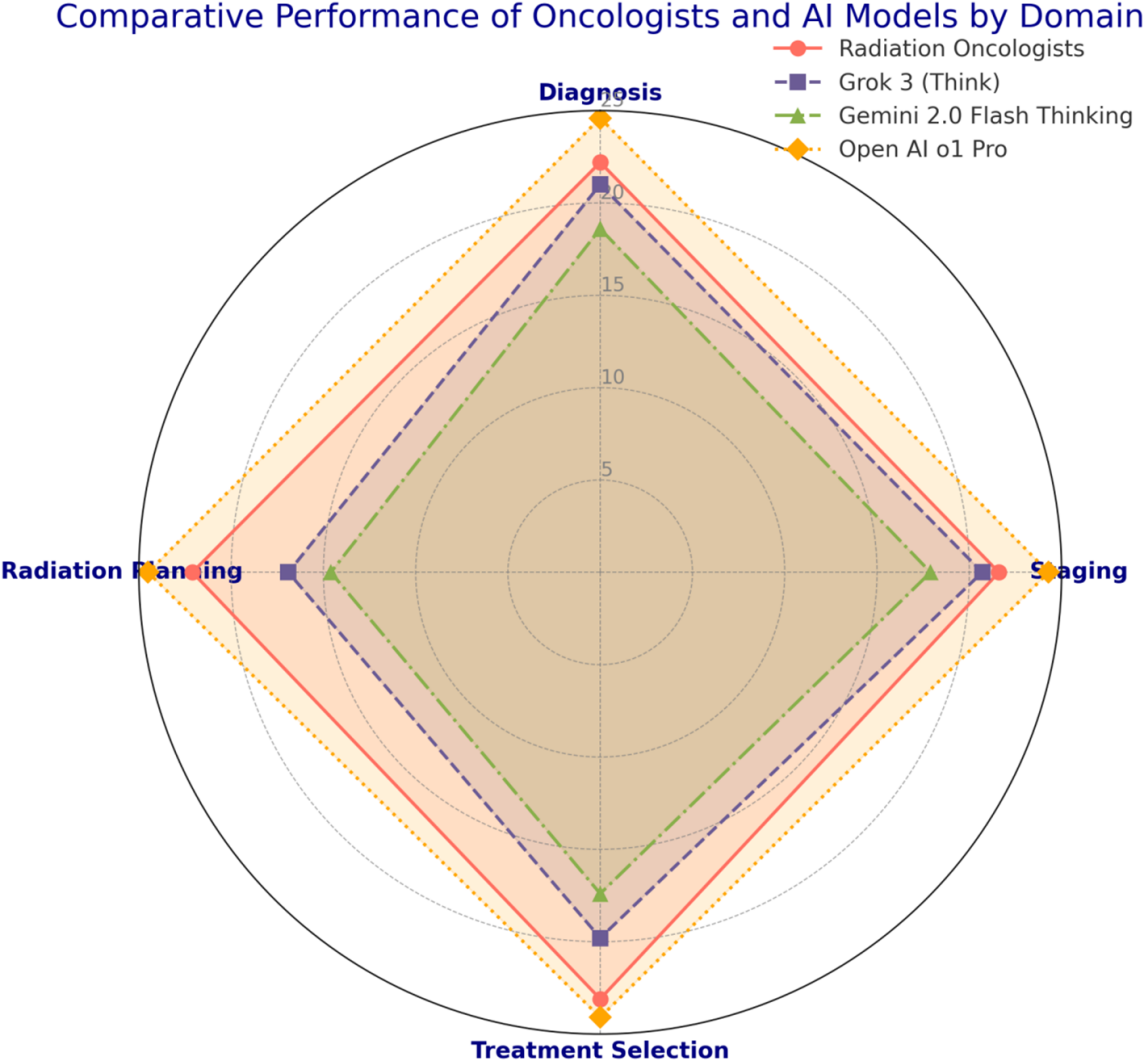
Comparative performance of oncologists and Al models by domain.

All 50 oncologists completed the guideline-based clinical examination, each providing one set of open-book answers. Three AI models—Grok 3 (Think), Gemini 2.0 Flash Thinking, and Open ai o1 pro—were each tested on the same 20 case-based questions in 10 separate chat instances, yielding 30 AI-generated answer sets. The final score of each set was determined by discarding the highest and lowest of the 12 experts’ ratings per question and averaging the remaining values. **Table 2** and **Figure 1** summarizes the total exam score distribution across human and AI cohorts. Open ai o1 pro demonstrated the highest mean total score (98.0 ± 1.9) among all participants and AI models, followed by radiation oncologists (91.5 ± 3.2). Grok 3 (Think) and Gemini 2.0 Flash Thinking achieved mean total scores of 82.3 ± 2.1 and 74.2 ± 3.4, respectively. The Kruskal–Wallis H test indicated a statistically significant difference in total scores among the four groups (p < 0.001). *Post hoc* pairwise comparisons revealed that Open ai o1 pro significantly outperformed each of the other three groups (p < 0.001).

**Table 2:** Distribution of total scores (out of 100) for human and AI groups.

Group	n	Mean ± SD	Median (IQR)	Range
Radiation Oncologists	50	91.5 ± 3.2	91 (89–94)	85–97
Grok 3 (Think)	10	82.3 ± 2.1	82 (80–84)	79–85
Gemini 2.0 Flash Thinking	10	74.2 ± 3.4	74 (72–77)	70–78
Open ai o1 pro	10	98.0 ± 1.9	98 (97–99)	95–100

### 3.3 Domain-specific performance

**Figure 2:**
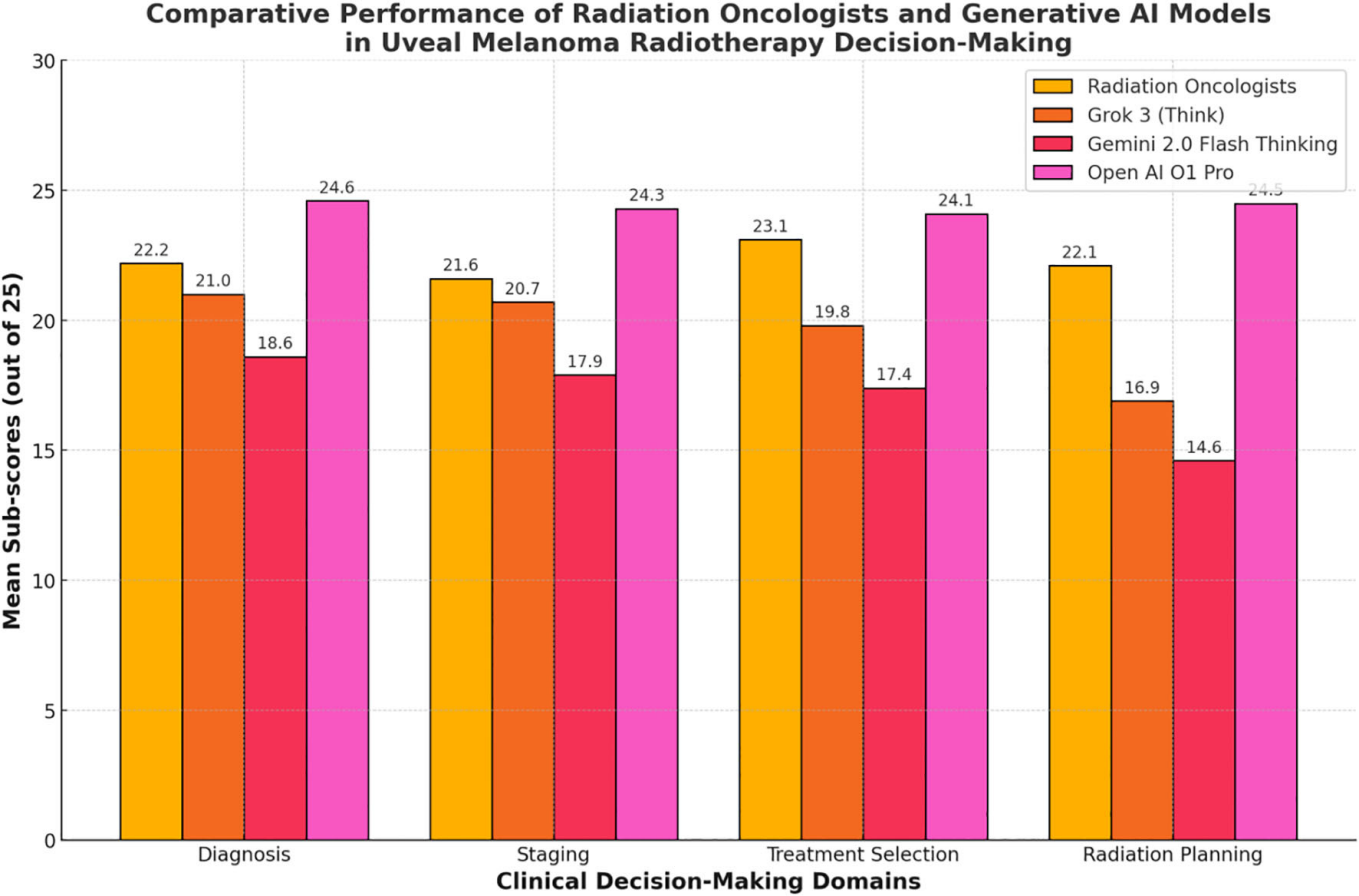
Comparative performance of radiation oncologists and generative Al models 30 in uveal melanoma radiotherapy decision-making.

Each of the 20 questions pertained to a specific domain of uveal melanoma radiotherapy (diagnosis, tumor staging, treatment selection, radiation planning). Scores within these domains were analyzed to highlight strengths and weaknesses across participant groups. **Table 3** and **Figure 2** shows the domain-specific sub-scores for each group. Radiation oncologists scored highest in treatment selection (mean = 23.1 ± 1.6 out of 25), aligning with their clinical experience. Grok 3 (Think) and Gemini 2.0 Flash Thinking achieved their best results in diagnosis and staging (both exceeding 80% of possible points in those two domains). Open ai o1 pro maintained near-maximum performance across all domains, consistently exceeding 95% of the points available.

**Table 3:** Domain-specific sub-scores (out of 25) for human and AI groups.

Domain	Radiation Oncologists (n=50)	Grok 3 (Think) (n=10)	Gemini 2.0 (n=10)	Open ai o1 pro (n=10)
Diagnosis	22.2 ± 2.1	21.0 ± 1.9	18.6 ± 2.2	24.6 ± 0.9
Staging	21.6 ± 2.3	20.7 ± 1.5	17.9 ± 2.4	24.3 ± 1.1
Treatment Selection	23.1 ± 1.6	19.8 ± 1.7	17.4 ± 3.3	24.1 ± 0.8
Radiation Planning	22.1 ± 2.0	16.9 ± 3.0	14.6 ± 2.7	24.5 ± 0.6

### 3.4 Time required for exam completion

All 50 radiation oncologists took an open-book exam with an upper time limit of 5 hours; they used an average of 4 hours 52 minutes (SD = 14 minutes). By contrast, the three AI models each completed the entire exam set within seconds to minutes. **Table 4** indicates the total response time for the 10 independent answer sets from each AI. Open ai o1 pro required a mean of 266 seconds to finalize one complete set of 20 answers, whereas Grok 3 (Think) and Gemini 2.0 Flash Thinking completed their answers in 92 seconds and 85 seconds, respectively. Human participants remained the slowest, though the difference in time was anticipated due to the complexity of referencing clinical guidelines and the open-book approach. English-as-second-language analysis. IELTS/TOEFL scores did not predict total accuracy (r = 0.12, p = 0.37) or completion time (r = -0.09, p = 0.48). Bland–Altman inspection revealed no systematic bias across the proficiency spectrum, alleviating concerns of residual linguistic confounding.

**Table 4:** Completion time for radiation oncologists and AI models.

Group	Mean Completion Time	Range
Radiation Oncologists (n=50)	4 h 52 min (± 14 min)	3 h 55 min–5 h 00 min
Grok 3 (Think) (n=10)	92 s (± 12 s)	75–105 s
Gemini 2.0 Flash Thinking (n=10)	85 s (± 15 s)	68–102 s
Open ai o1 pro (n=10)	266 s (± 23 s)	240–298 s

### 3.5 Inter-rater reliability and score variability

Twelve recognized experts each graded the 80 anonymized sets (50 from oncologists, 30 from AI models). Fleiss’ Kappa among the 12 experts was 0.89 (95% CI: 0.86–0.91), reflecting excellent inter-rater agreement. Following the scoring protocol, the highest and lowest scores from each of the 12 examiners were discarded for each answer, and the remaining 10 ratings per question were averaged to produce the final question-level result. **Table 5** demonstrates the Fleiss’ Kappa values by question domain, indicating consistently high levels of agreement.

**Table 5:** Fleiss’ Kappa for expert grading by domain.

Domain	Fleiss’ Kappa (95% CI)
Diagnosis	0.90 (0.86–0.93)
Staging	0.88 (0.84–0.91)
Treatment Selection	0.87 (0.83–0.90)
Radiation Planning	0.89 (0.85–0.92)

### 3.6 Statistical comparisons across groups

Given the nonparametric distributions observed in some domain scores, the Kruskal–Wallis H test was used to compare median performance. Radiation oncologists, Grok 3 (Think), Gemini 2.0 Flash Thinking, and Open ai o1 pro formed four groups. **Table 6** presents the results of the Kruskal–Wallis H test and subsequent *post hoc* analysis for total scores and domain sub-scores. There were significant differences among the four groups in both overall scores and each sub-domain (p < 0.001). Pairwise *post hoc* comparisons confirmed that Open ai o1 pro scored significantly higher (p < 0.001) than all other participants in all domains. Moreover, radiation oncologists significantly outperformed Grok 3 (Think) and Gemini 2.0 Flash Thinking in every domain except for diagnosis and staging (p < 0.05). Grok 3 (Think) had higher median scores than Gemini 2.0 Flash Thinking (p < 0.05).

**Table 6:** Kruskal–Wallis H test and *post hoc* analysis for total and domain scores.

Variable	H Statistic (df=3)	p-value	*Post Hoc* Comparisons (p < 0.05)
Total Score	39.24	<0.001	Open ai o1 pro > Oncologists > Grok 3 > Gemini 2.0
Diagnosis	25.17	<0.001	Open ai o1 pro > Oncologists ≈ Grok 3 > Gemini 2.0
Staging	27.48	<0.001	Open ai o1 pro > Oncologists ≈ Grok 3 > Gemini 2.0
Treatment Selection	36.92	<0.001	Open ai o1 pro > Oncologists > Grok 3 > Gemini 2.0
Radiation Planning	42.1	<0.001	Open ai o1 pro > Oncologists > Grok 3 > Gemini 2.0

All comparisons confirm that Open ai o1 pro achieved the highest performance, followed by the radiation oncologists, Grok 3 (Think), and finally Gemini 2.0 Flash Thinking. The complete distribution of domain-specific scores is consistent with the total score ranking. No contradictory outcomes were identified in any subgroup analysis.

## 4 Discussion

In many clinical contexts, experienced physicians are widely seen as a cornerstone of consistent patient outcomes, yet the present findings that Open ai o1 pro exceeded the already high performance of experienced radiation oncologists in the final scores dovetail with emerging evidence that certain AI systems can sometimes match or even surpass expert-level human decision-making. These observations are reminiscent of similar patterns noted in other highly specialized domains, where advanced computational models have rapidly outpaced many traditional benchmarks. In this study, however, the radiation oncologists nonetheless achieved strong overall scores, reinforcing the idea that extensive clinical training and real-world experience remain indispensable even when facing formidable AI counterparts. From a practical standpoint, the findings strengthen the case that even when an algorithm outperforms clinicians on a written test, human oversight remains mandatory to verify provenance of recommendations, individualized care, and assume ethical–legal responsibility for final treatment decisions.

Our study found that radiation oncologists excelled in the selection of appropriate treatments, scoring consistently high in areas such as radiotherapy modality choice and individualized patient care. This tendency for elevated performance in hands-on clinical judgment is consistent with prior research, which underscores the unique contributions of clinical experience—particularly when nuanced patient factors, like comorbidities and personal treatment preferences, might not always be fully captured by algorithmic approaches (41–45). Although Open ai o1 pro attained near-perfect accuracy, the oncologists’ balanced decision-making in complex scenarios, especially when selecting treatments based on established protocols for borderline or large tumors, reflects the distinct advantage of their practical familiarity with challenging cases.

In this study, Grok 3 (Think) and Gemini 2.0 Flash Thinking demonstrated moderate to high accuracy in diagnosis and staging, offering particularly consistent results on questions involving early detection markers and imaging-based classification. Their performance suggests that many AI models, when properly trained, can carry out reliable domain-specific analyses, especially in systematically structured tasks like tumor measurements and classification criteria. Notably, Grok 3 (Think) showed higher scores than Gemini 2.0 Flash Thinking, indicating that incremental improvements in algorithmic architecture or training approaches may account for differences in performance among even closely related models. Despite these variations, it is noteworthy that both AI systems occasionally lagged behind the human cohort in more intricate radiation planning details or in addressing complex patient comorbidities. An additional factor contributing to the marked time gap is the fundamentally different workflow: clinicians needed to search, open, and cross-reference guideline PDFs or institutional protocols before composing free-text answers, whereas the language models had the relevant knowledge already encoded. This retrieval step, although slower, mirrors real-world practice where guideline consultation is a safety check rather than wasted effort.

We did not find that the remarkably short time frames in which generative AI systems formulate their responses diminished the credibility or accuracy of their final solutions. While the oncologists completed the exam in several hours, the three AI models arrived at answers in under five minutes. In many technology-driven sectors, there are concerns that computational speed might sacrifice thoroughness, but these models have showcased an ability to synthesize vast amounts of reference data with surprising agility. Even with unrestricted access to guidelines and reference texts, the human participants took considerably longer, although the open-book environment did not notably diminish their accuracy. It remains unclear whether more time necessarily equates to better performance, and future research might probe whether certain real-time AI-driven insights could actually refine human decision-making by providing rapid second opinions. Because generative AI produced high-quality answers in under five minutes, the saved time could be reallocated to patient-facing tasks. In routine practice, clinicians could use the extra hours for longitudinal counselling about visual-function expectations or for multidisciplinary tumor-board deliberations, potentially leading to more holistic care without extending clinic schedules. Radiation-planning questions posed the greatest challenge for Grok 3 and Gemini 2.0. Both models struggled with margin-expansion conventions for plaque placement and with accounting for dynamic ocular rotations during proton-beam set-up—nuances that experienced planners internalize through hands-on contouring. These specific pitfalls highlight priority areas for future model fine-tuning. The present cohort practices in mixed−modality institutions where plaque and proton facilities coexist. In regions limited to proton beam therapy (e.g., many Nordic countries) or, conversely, centers that rely exclusively on plaque brachytherapy, the absolute scores may shift but the relative human−versus−AI margin is likely to persist because the question set emphasized dose−constraint logic and anatomical considerations common to both modalities. Future validation should nevertheless replicate our protocol in single−modality environments to confirm transferability. Clinicians completed an open−book exam with unrestricted access to current guidelines and textbooks, whereas the AI models relied on frozen internal weights. Although this asymmetry appears advantageous to humans, it mirrors real−world practice in which oncologists routinely consult references during planning, while deployed AI assistants will draw on static (but audit−checked) corpora. Allowing both parties to operate under their native knowledge−retrieval paradigms therefore offers a fair comparison of how each would perform in everyday workflows rather than in an artificially constrained setting. Expert graders flagged that all three AI systems mentioned cataract formation, radiation retinopathy, and neovascular glaucoma in ≥80 % of relevant answers, but only Open ai o1 pro consistently provided guideline−level dose thresholds (e.g., 50 Gy EQD2 to fovea) and follow-up intervals. Grok 3 and Gemini 2.0 occasionally omitted the need for monthly fundoscopy in the first post−treatment year, indicating a residual gap in long−term toxicity counseling that warrants model fine-tuning. Differences in performance are likely rooted in heterogeneity of training data and architectural emphasis. Open ai o1 pro’s medical-alignment phase includes curated radiotherapy protocols and dose-planning textbooks, whereas Gemini 2.0 relies more heavily on general-domain web data, and Grok 3 emphasizes peer-reviewed publications. Such contrasts influence the models’ grasp of anatomical dose constraints, explaining the gradient we observed across radiation-planning items. Cost−effectiveness merits explicit mention. In our setting, Open ai o1 pro processed a 20−case bundle in ≈4.4 min—about 0.073 GPU−hours. Even on an on−demand H100 instance priced at USD 3 per GPU−hour, the raw compute cost is ≈USD 0.22, bringing the total inference expense (compute + platform fee) to ≈USD 1.90. A median 4.9 h of consultant time at ¥437 per hour (≈USD 60) totals ≈USD 294, yielding a wage−adjusted cost differential of roughly 155−fold in favor of the AI workflow. We define expert−in−the−loop as a pipeline in which the AI drafts a recommendation that is mandatorily reviewed—and signed off—by a subspecialist before being entered into the electronic medical record. In an AI−first workflow, the model generates the initial plan that is automatically populated into the planning system, with human review occurring only when predefined safety triggers (e.g., optic−nerve Dmax > 55 Gy) are violated. The former emphasizes augmentation, the latter automation; our data support the expert−in−the−loop model as the safer near−term path in ocular oncology. Even after accounting for double-checking (15 min, USD 16), the time-saving-to-cost ratio is roughly 18:1, indicating that selective AI assistance in routine ocular-oncology planning could yield substantial economic dividends while freeing clinicians for direct patient counselling.

Our study has the advantage of an extensive recruitment of radiation oncologists, each backed by over a decade of professional experience and significant academic credentials. By ensuring that all participants completed a uniform 15-day review course, we minimized disparities in baseline knowledge and thus bolstered the reliability of the human cohort’s performance. The detailed, guideline-based clinical examination also captured multiple critical domains—diagnosis, tumor staging, treatment selection, and radiation planning—offering a comprehensive assessment of both human and AI capabilities. The double-blinded grading procedure, wherein transcribed answers were anonymized before scoring by twelve recognized experts, further bolstered the objectivity and methodological rigor of this work. The present study also has several limitations. Although the questions encompassed key aspects of uveal melanoma radiotherapy, no single examination can fully reflect every subtlety encountered in clinical practice. The open-book format, while consistent with modern reference-driven workflows, may not simulate the stress and resource constraints typically found in actual treatment decision-making environments. Additionally, we relied on three high-profile AI models that were readily available during the study period; the technology landscape, however, is evolving rapidly, and subsequent generations of these models—or entirely new AI platforms—may demonstrate different performance patterns. It would also have been interesting to explore deeper interactions between humans and AI models working in tandem, a scenario that is increasingly plausible in real-world oncology settings.

## 5 Conclusion

This study provides robust evidence that advanced generative AI models, particularly Open ai o1 pro, can excel in clinical decision-making tasks for uveal melanoma radiotherapy, surpassing the high baseline performance of experienced radiation oncologists across all examined domains. Despite the near-perfect accuracy and extraordinary speed demonstrated by Open ai o1 pro, the human experts maintained strong overall scores—most notably in treatment selection, where practical familiarity with nuanced patient factors remains crucial. The other two AI models, Grok 3 (Think) and Gemini 2.0 Flash Thinking, performed moderately well, suggesting that architecture and training differences affect AI effectiveness even within the same domain. While open-book conditions and the focus on selected guideline-based questions may not fully replicate the complexities of real-world practice, the consistently high inter-rater reliability underscores the validity of these findings. Future research should investigate how clinicians and AI might optimally collaborate, particularly in borderline or complex cases, to improve both the speed and quality of uveal melanoma care while preserving critical human insights.

## Data Availability

The original contributions presented in the study are included in the article/supplementary material. Further inquiries can be directed to the corresponding author.

## Appendix Table 1. Guideline-based clinical examination for uveal melanoma radiotherapy.

**Table:**

No.	Clinical Scenario	Question	Domain	Points
1	A 55-year-old male presents with a pigmented choroidal lesion measuring 2 mm in height and 8 mm in basal diameter. On B-scan ultrasonography, the lesion demonstrates low-to-medium internal reflectivity. Fundoscopy suggests subtle orange pigmentation on its surface.	Which additional diagnostic imaging modalities or clinical evaluations are most important to confirm a diagnosis of uveal melanoma, and how do they help differentiate benign from malignant lesions? Provide the rationale based on guidelines.	Diagnosis	5
2	A 42-year-old female with recent onset of floaters and mild visual disturbances undergoes slit-lamp biomicroscopy, which reveals a small, pigmented iris lesion suspicious for melanoma. There is no evidence of hyphema.	Describe the recommended workup steps to confirm the diagnosis of iris melanoma and rule out other differential diagnoses. Include key findings that would raise concern for malignant transformation according to consensus guidelines.	Diagnosis	5
3	A 60-year-old patient reports gradual peripheral vision loss over 6 months. A fundus exam shows a dome-shaped elevated lesion in the choroid. Ultrasound confirms a medium reflectivity lesion. No extraocular extension is noted.	Based on standard clinical guidelines, what are the primary risk factors and clinical/red-flag signs that strongly suggest uveal melanoma rather than a choroidal nevus? How should these signs be systematically assessed and documented?	Diagnosis	5
4	A 51-year-old male with a flat, lightly pigmented lesion in the posterior pole is referred by an optometrist for possible early choroidal melanoma. The lesion is <2 mm in thickness with minimal subretinal fluid.	Outline the recommended follow-up intervals and the critical diagnostic thresholds that would indicate progression from a suspicious nevus to a treatable uveal melanoma.	Diagnosis	5
5	A 65-year-old female has a pigmented choroidal lesion measuring 3 mm thickness and 10 mm in diameter but is asymptomatic. Fundus photography shows drusen over the lesion.	Discuss the role of multimodal imaging and the “monitor *vs*. treat” criteria in borderline lesions according to current guidelines. What diagnostic factors determine whether immediate intervention is required?	Diagnosis	5
6	A 56-year-old patient is newly diagnosed with uveal melanoma. Basal diameter measures 12 mm, and tumor thickness is 6 mm. The patient has no systemic symptoms, and blood tests are within normal limits.	Using the AJCC classification for uveal melanoma, determine the T category and explain the staging process. Which additional investigations are recommended to complete staging?	Staging	5
7	A patient with a 7-mm thick choroidal melanoma underwent orbital MRI that showed no scleral or extraocular extension, but borderline involvement of the ciliary body could not be ruled out.	According to standard staging guidelines, explain how even minimal ciliary body involvement may alter T staging and the overall approach to management. What imaging or diagnostic steps could further clarify the extent of ciliary body involvement?	Staging	5
8	A 60-year-old patient has a choroidal tumor measuring 9 mm thickness and 14 mm in basal diameter, with mild subretinal fluid and no metastasis on systemic workup.	Classify the tumor by T category (AJCC or COMS-based) and discuss the potential significance of subretinal fluid in staging. How does subretinal fluid factor into local tumor extent classification in the current guidelines?	Staging	5
9	A 59-year-old patient with a large choroidal melanoma (11 mm thick, 17 mm basal diameter) complains of intermittent eye pain. PET-CT scan reveals no evidence of distant metastases, but there is mild extraocular extension posteriorly.	According to the AJCC classification, how would this be staged (T category and overall stage)? Explain how the presence of extraocular extension influences staging and prognosis, and which additional clinical evaluations might be indicated.	Staging	5
10	A 45-year-old patient with a medium-sized uveal melanoma undergoes thorough imaging. The tumor abuts the optic disc with partial involvement of the macula, but remains confined to the globe on MRI.	Discuss the relevance of tumor location in staging (including involvement of critical structures like the optic disc or macula) and how these anatomic details factor into risk stratification and prognosis. Which official staging classification elements specifically account for tumor location?	Staging	5
11	A 52-year-old patient with a 5-mm thick choroidal melanoma and good visual acuity (20/30) is weighing treatment options. The lesion is located near the macula.	Evaluate the recommended treatment strategies for a small to medium choroidal melanoma near the macula. Which factors guide the choice of eye-conserving radiotherapy versus alternative approaches? Cite relevant guideline recommendations.	Treatment Selection	5
12	A 63-year-old patient presents with a medium-sized choroidal melanoma that is also amenable to either plaque brachytherapy or proton beam therapy. The patient is uncertain about preserving vision.	Compare the key considerations—effectiveness, vision outcomes, complication profiles, and patient preferences—that inform the selection between plaque brachytherapy and proton beam therapy. Summarize the evidence base that helps guide clinical decisions.	Treatment Selection	5
13	A patient with a large choroidal melanoma (10 mm thick, 15 mm diameter) expresses a strong desire to preserve the globe if possible. Systemic evaluation shows no metastatic disease.	Discuss the role of radiotherapy in large tumors, including possible combination with local resection or other interventions. What guidelines or consensus recommendations address borderline or large tumors that pose a higher risk of local failure or significant ocular morbidity?	Treatment Selection	5
14	A 58-year-old patient has a 4-mm thick iris melanoma. Visual acuity remains excellent, and there is minimal tumor extension to the trabecular meshwork.	Outline the standard treatment approaches for iris melanoma, differentiating indications for brachytherapy, proton therapy, or local resection. How do guidelines suggest balancing tumor control with preservation of visual function, especially in anterior segment tumors?	Treatment Selection	5
15	A 69-year-old patient with a uveal melanoma recently experienced elevated intraocular pressure (IOP). The tumor measures 8 mm thick.	Describe treatment selection considerations when IOP is affected by the tumor. How do guidelines suggest addressing ocular hypertension while planning definitive radiotherapy?	Treatment Selection	5
16	A 55-year-old with a 7-mm thick choroidal melanoma is scheduled for plaque brachytherapy. The tumor is located 2 mm from the optic nerve head.	Discuss key dosimetric considerations and how guidelines recommend balancing adequate tumor coverage with sparing of critical structures.	Radiation Planning	5
17	A 47-year-old patient requires proton beam therapy for a 5-mm thick choroidal melanoma in the macular region.	Summarize the standard steps in proton beam planning for uveal melanoma, including tumor localization, immobilization, and margin expansions. What are the critical organs-at-risk, and how do guidelines advise minimizing dose to these structures?	Radiation Planning	5
18	A patient with a large choroidal melanoma (9 mm thick) near the ora serrata is a candidate for stereotactic radiosurgery.	Outline the contouring guidelines and dose constraints for stereotactic radiosurgery in uveal melanoma. Which imaging modalities help ensure accurate delineation of tumor boundaries, and how do guidelines recommend accounting for potential movement of the eye?	Radiation Planning	5
19	A 62-year-old patient with a choroidal melanoma underwent plaque brachytherapy three months ago and returns with new onset of radiation retinopathy symptoms.	Discuss the recommended follow-up schedule and typical timeline for detecting radiation complications. How do guidelines suggest managing or mitigating these post-treatment adverse events?	Radiation Planning	5
20	A 68-year-old patient with a history of diabetes and moderate cataract is about to receive proton beam therapy for a posterior choroidal melanoma.	Explain how comorbid conditions might influence radiotherapy planning and follow-up care to preserve visual function. What consensus-based modifications or precautions are recommended to mitigate complications?	Radiation Planning	5
